# Body Weight and Allergic Asthma: A Narrative Review

**DOI:** 10.3390/jcm13164801

**Published:** 2024-08-15

**Authors:** Ikuyo Imayama, Jacob D. Eccles, Christian Ascoli, Elizabeth Kudlaty, Gye Young Park

**Affiliations:** 1 Division of Pulmonary, Critical Care, Sleep and Allergy, University of Illinois Chicago, Chicago, IL 60612, USA; jeccle2@uic.edu (J.D.E.); cascoli@uic.edu (C.A.); ekudlaty@uic.edu (E.K.); parkgy@uic.edu (G.Y.P.); 2 StatCare, Knoxville, TN 37919, USA

**Keywords:** allergic asthma, type 2 phenotype, excess body weight, obesity

## Abstract

Obesity is a known risk factor for asthma development, progression, and exacerbation. Nevertheless, the underlying pathophysiological mechanisms explaining how obesity contributes to the development and progression of asthma have yet to be established. Here, we review human studies examining the associations between asthma and obesity, focusing on the literature from the past 10 years. Overall, current evidence suggests that while both asthma and obesity are complex diseases with significant heterogeneity, they both share various features of chronic inflammation. Furthermore, the interactions between asthma and obesity likely involve allergen-specific T helper type 2 (type 2) immune responses, as well as diverse non-type 2 inflammatory pathways. However, despite considerable progress, studies to date have not definitively elucidated the mechanisms that account for the observed association. A large-scale population-based study combined with translational immunological research, including targeted asthma therapies and pharmacological weight loss therapies, may be required to properly dissect the details of obesity-related asthma pathophysiology.

## 1. Introduction

Asthma is a reversible obstructive airway disease caused by a hyperactive response triggered by an inhaled stimulus. Historically, asthma has been classified as allergic or non-allergic; allergic asthma is considered to develop following exposure to an allergen, sensitization to it, and expansion of allergen-specific T helper type 2 cells (type 2) through recurrent exposure [1]. However, more recent evidence has revealed that asthma is not a simple allergic reaction to an allergen but rather a complex interaction of chronic immunologic and inflammatory processes [2,3]. Given significant heterogeneity, asthma continues to be subclassified by phenotype. In a nuanced revision of the allergic/non-allergic dichotomy mentioned above, asthma may be more precisely categorized into type 2-high and type 2-low based on the immunologic mechanisms found to be contributing. Type 2-high and -low phenotypes are important in clinical practice because they guide asthma treatment [4]. In particular, the type 2-high phenotype is more responsive to corticosteroids and is often amenable to additional biologic treatments for uncontrolled cases [5]. Common features of type 2-high asthma are early onset, elevated allergen-specific immunoglobulin E (IgE), and responsiveness to corticosteroid therapy. In comparison, the common features of type 2-low asthma are later onset, female sex, normal IgE levels, and resistance to corticosteroid therapy [6]. 

The World Health Organization defines obesity as “a complex disease defined by excess fat deposits that can impair health”. Body mass index (BMI)—weight (kg) divided by height (m)^2^—greater than or equal to 30 is commonly used for its diagnosis. Epidemiologic studies have demonstrated that obesity is associated with an increased risk of allergic diseases, including allergic rhinitis, asthma, and atopic dermatitis [7]. Further studies have elaborated that obesity induces immunological changes resulting in decreased tolerance to exogenous antigens and skewing of the immune response toward a type 2 profile, increasing the risk of allergy and other immune-mediated disease [8]. However, perhaps in contrast to these reports, other epidemiological studies have shown that obesity has an even stronger association with the type 2-low asthma phenotype [9,10]. Obesity is known to contribute to chronic inflammation involving the activation of M1 macrophages and CD8+ T lymphocytes, which have been shown to be capable of producing a cascade of non-type 2 inflammatory mediators, including interleukin (IL)-1β, IL-6, interferon (IFN)-γ, and tumor necrosis factor (TNF)-α [11]. Thus, obesity presents somewhat of a paradox, given that it simultaneously correlates with both type 2-low asthma and the full spectrum of allergic diseases, including type 2-high asthma. 

**Figure 1:**
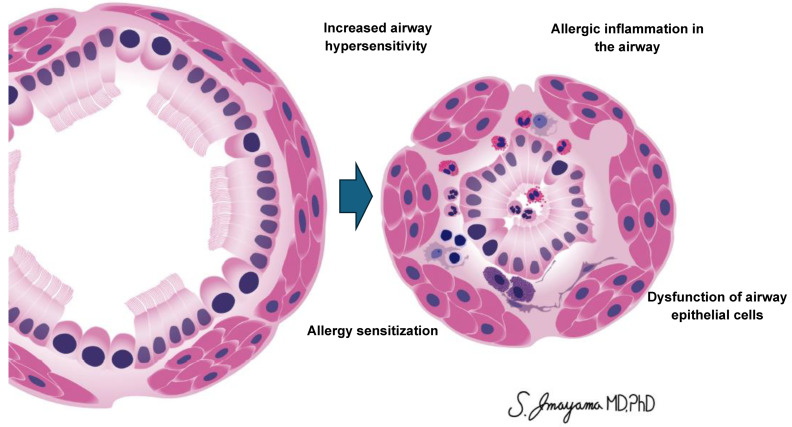
Summary of mechanisms linking obesity to asthma discussed in this article.

As an additional wrinkle, longitudinal studies examining patients with severe asthma have shown that a phenotypic transition from type 2-high to type 2-low often occurs over time, with the most severe asthmatics generally exhibiting elevated eosinophilic inflammation and high type 2 biomarkers throughout 10 years at some point in their disease, though these signals may eventually wane in transition to a type 2-low phenotype [12,13,14]. Based on these reports, it is possible that type 2-high and type 2-low asthma may be interchangeably present as the dominant phenotype during various disease periods. As such, a simple dichotomous division of type 2-high and type 2-low phenotypes may not be sufficient to categorize asthma, and thorough classification should be inclusive of additional cellular/molecular pathways [6]. Thus, while accumulating evidence suggests that the increased inflammation seen in obesity could play a significant role in asthma, the precise mechanisms have yet to be established. This review discusses how obesity impacts asthma by reviewing published human studies primarily within the past 10 years (Figure 1).

The literature search was performed using the following databases: (1) PubMed, (2) Google Scholar, (3) Google, (4) Clinical Key, and (5) Ovid. Cross-referencing was also used to identify the literature. The following keywords were used: human, asthma, allergic asthma, allergy, hyperreactivity, bronchial provocation, methacholine challenge test, excess body weight, obesity, weight gain/loss, inflammation, helper T cell, CD4+ T cells, airway epithelium, eosinophils, innate lymphoid cells, alarmins, and fractioned exhaled nitric oxide (FeNO). All papers were reviewed independently of the study design. The papers were reviewed by the first author for their appropriateness. If there was any cause for concern about a given study, an additional author provided a secondary review. All authors contributed to verifying the papers referenced in this manuscript for relevance. 

## 2. Are Obese People More Prone to Allergen Sensitization? 

Multiple studies have shown that excess body weight is correlated with allergic disease in children. In a meta-analysis of 42 individual studies, Wooldridge and colleagues showed that a 1-kg increase in birth weight was associated with a 44% increased risk of food allergy and a 17% increased risk of atopic dermatitis [15]. In another study of 707 pre-pubertal children (aged 3–10 years), obese status was associated with a 72% increased risk of allergic asthma and/or rhinitis compared to average weight (odds ratio [OR] 1.71, 95% confidence interval [CI] 1.08–2.71) [16]. Despite the established connection between obesity and clinical allergic conditions, evidence for a specific mechanism is inconsistent. For example, a birth cohort of 2075 infants, followed for 8 years, showed that higher BMI at ages 1 and 7 was associated with elevated serum IgE levels to inhaled allergens [17]. However, a follow-up study by Lucas and colleagues showed no association between body weight and specific allergen sensitization in 125 children aged 1 to 16 [18]. In another study in Poland including 2133 adults aged 20–44, despite increased asthma prevalence in overweight and obese adults, there was no significant difference in frequency of allergic sensitization, as evaluated using a skin prick test (SPT), to 15 aeroallergens, across normal, overweight, and obese groups [19]. 

Meanwhile, several studies have demonstrated variation in SPTs across other demographic modifiers. In a longitudinal cohort of the general population (1439 children and adolescents, 9844 adults), the prevalence of positive SPTs to at least one aeroallergen was higher in men in almost all age groups except 10–15 years old, with SPT positivity peaking at 10–15 years in women and 19–29 years in men [20]. Additionally, childhood status, higher socioeconomic status, higher eosinophil count, and never smoking were associated with an increased likelihood of a positive SPT [20]. Adding another layer of complexity, in a 15-year longitudinal study of 3409 adults (age 31 to 46), 243 changed from negative to positive SPTs while 196 changed from positive to negative SPTs [21]. It should also be noted that several studies have demonstrated longitudinal inconsistency in SPT and serum IgE testing, with results known to be influenced by numerous patient factors [21,22,23]. Furthermore, changes in aeroallergen sensitization patterns over time may reflect distinct physiological processes in adults and children. In clinical practice, allergy testing is repeated at 2-year intervals in children vs. 10-year intervals in adults. Based on the literature we reviewed, the associations between allergen sensitization and body weight are yet to be established. 

## 3. Are Obese People More Prone to Airway Hyperreactivity? 

Airway hyperreactivity triggered by smooth muscle contraction is a key feature of asthma. In one study in 697 adults living in Tasmania, a higher BMI was associated with increased airway hyperreactivity in non-asthmatics and well-controlled asthmatics, but not in actively symptomatic asthmatics [24]. With respect to the latter observation, one might consider that the lack of a differential response in active asthmatics may reflect a reduced dynamic range of contraction when the airway has already contracted due to active disease. 

An in vitro study compared airway smooth muscle cells derived from non-obese (N = 14) and obese (N = 15) human subjects. Cells from obese subjects demonstrated augmented intracellular calcium flux, increased phosphorylation of myosin light chain, and more powerful contraction upon agonist delivery, compared with cells from the non-obese subjects, suggesting increased airway reactivity [25]. An additional mechanistic study probed the interaction between human adipose tissue and airway smooth muscle cells using conditioned media from explanted adipocytes [26]. The adipocyte-conditioned media did not alter smooth muscle cell proliferation or migration; however, glycolysis was found to be augmented. Subsequent inhibition of glycolysis reduced myosin light chain phosphorylation, suggesting that altered glucose metabolism may mediate airway hyperreactivity in obesity [27]. Another study investigated changes in nasal lavage fluids after intranasal challenge with house dust mite extract in lean (BMI < 25 kg/m^2^) and obese (BMI ≥ 30 kg/m^2^) individuals with allergic rhinitis and asthma who had a positive SPT to dust mite [28]. The study provided evidence of increased production of H_2_O_2_, IL-33 (a type 2-inducing alarmin), and IL-13 (type 2-induced cytokine), in obese asthmatics as compared with lean asthmatics, suggesting an augmented response to the allergen in obese subjects. Type 2 cytokines generally, including IL-4, are thought to play a key role in triggering airway hyperreactivity [29], and an additional study of 86 asthmatic adults showed that serum IL-4 was significantly increased in overweight and obese asthmatics compared with their average-weight counterparts [30]. 

Interventional studies investigating the effects of weight loss have reported that weight loss may improve airway hyperreactivity. In one study of 27 obese asthmatics undergoing bariatric surgery, 89% had positive provocation tests at baseline, and this fell to 46% at 3 months, 45% at 6 months, and 52% at 12 months post-operatively [31]. In a cohort study of obese women with (N = 11) and without (N = 15) asthma followed for 12 months after bariatric surgery, the dose of methacholine required for bronchoprovocation increased significantly from 3.42 mg/mL at baseline to 9.94 mg/mL at 12 months, indicating reduced airway hyperreactivity [32]. Furthermore, visceral fat leptin expression was found to be negatively associated with the provocation dose of methacholine (Spearman’s *ρ* = −0.8; *p* < 0.01), again suggesting adiposity contributing to airway reactivity [32]. In a prospective, controlled, parallel-group study testing the impact of a 3-month behavioral weight loss program in 22 obese asthmatics, there was also a significant increase in bronchoprovocation methacholine dose (5.02 mg/mL to 10.2 mg/mL) at 3 months [33]. It should be noted, however, that in another study evaluating 22 obese, severe asthmatics undergoing weight loss surgery, there was no change in the provocation dose of methacholine, FeNO level, sputum cell analysis (eosinophils, neutrophils, lymphocytes, macrophages, and epithelial cells), or serum IgE at 6 months after surgery [34]. Thus, although not all studies have reported a significant association between body weight and airway hyperreactivity, the current literature overall suggests a positive correlation, where weight loss may reduce airway hyperresponsiveness.

## 4. Do Obese People Increase Allergic Inflammation in the Airway?

Eosinophils are known to play a pivotal role as effectors in the type 2 pathway, and studies have used eosinophil counts from various locations, including blood, sputum, bronchoalveolar lavage (BAL), and lung tissue, as surrogate markers of inflammation to study the interaction of obesity with asthma. Though frequently employed, it should be stated that many of these measurements correlate somewhat weakly with clinical readouts of asthma severity. Another popular measurement used to assess allergic inflammation in the airway is FeNO, the product of inducible nitric oxide synthase (iNOS) activity in airway epithelium stimulated by type 2 cytokines [35]. The following section reviews the literature correlating body weight with eosinophils in the blood and airways, type 2 innate lymphoid cells (ILC2), alarmins, and FeNO. 

### 4.1. Blood Eosinophils 

Notably, while airway eosinophils represent a more direct marker of airway inflammation, appropriate sample collection suffers from logistical complexity and unreliability, and is thus generally deferred in favor of blood sampling. Currently, the Global Initiative for Asthma (GINA) recommends that clinicians utilize blood absolute eosinophil count (AEC) to identify patients who are at risk of asthma exacerbations and likely to benefit from biologic therapy [4]. Providing validation for this approach, a meta-analysis of 23 studies (N = 155,772) concluded that AEC ≥ 200 cells/μL is significantly associated with the risk of asthma exacerbation with OR 1.31 (95% CI 1.16 to 1.49) [36]. Studies have also supported the diagnostic value of AEC by correlating it with eosinophils in the airways. In 336 adult-onset asthma patients, blood eosinophils and FeNO were shown to have comparable diagnostic accuracy in detecting elevated sputum eosinophils (≥3%), with the area under the curve 0.83 (95% CI 0.78–0.87) and 0.82 (95% CI 0.77–0.87), respectively, whereas total serum IgE was less accurate [37]. Another study also found that blood eosinophils (r = 0.59) and FeNO (r = 0.52) correlated with sputum eosinophils, whereas serum periostin failed to demonstrate an association (r = 0.09), suggesting again that blood eosinophils can be used as a surrogate marker for sputum eosinophils (and thus airway inflammation) in managing asthma patients [38].

Several studies have reported a positive correlation between body weight and AEC. A population-based cohort of 13,301 people reported positive associations between AEC and BMI [39], while a subsequent larger cohort confirmed this result independently in men and women (N = 44,035) [40]. However, in a subgroup of the National Health and Nutrition Examination Survey (NHANES, N = 789), obesity and high waist circumference were associated with lower AEC without significant differences in FeNO [41]. Given such discrepancy, a meta-analysis of observational studies was conducted that examined AEC in asthma (39 studies), severe asthma (12 studies), chronic obstructive pulmonary disease (23 studies), control (seven studies), and general populations (14 studies), reporting that higher AEC is positively associated with higher BMI [42]. Curiously, in a subgroup (N = 4650) of a Japanese community-based survey of 9789 people, AEC positively correlated with BMI, except for subjects with the highest quartile of eosinophils (≥200/μL), accounting for age, gender, smoking history, white blood cell count, and serum IgE level [43]. In the highest AEC quartile, BMI instead negatively correlated with eosinophil counts, possibly suggesting a non-linear association between body weight and AEC at the higher end of eosinophil levels. Finally, considering eosinophil function, another study in children and adolescents reported that peripheral blood eosinophil chemotaxis was increased in asthmatic obese individuals compared with asthmatic non-obese individuals [44]. Meanwhile, a study clarified that adipose-derived hormones could regulate eosinophil activity, with leptin stimulating and adiponectin attenuating measured adhesion and chemotaxis of eosinophils extracted from human peripheral blood [45,46]. 

### 4.2. Sputum and Submucosal Eosinophils 

Sputum eosinophils have also been studied to assess airway inflammation in asthma, although given that relatively few laboratories provide analysis, the assay is not widely used in routine clinical practice. Nevertheless, a meta-analysis of six studies concluded that there was a significant reduction in asthma exacerbations when treatment was guided by sputum eosinophil counts, as compared to clinical symptoms alone or with lung function tests, with pooled OR of 0.57 (95% CI 0.38 to 0.86) [47]. Perhaps complicating this result with respect to obesity, another study of 131 subjects with severe asthma found that while BMI positively associated with sputum IL-5 (eosinophil growth factor) and bronchial submucosal eosinophil counts, it did not correlate with sputum or blood eosinophil counts [48]. Van der Wiel and colleagues also examined the association of eosinophil counts in blood, sputum, and airway biopsy samples among patients with moderate to severe asthma [49]. In non-obese subjects, submucosal eosinophils were significantly associated with blood eosinophils (r = 0.232) and percentage of sputum eosinophils (r = 0.396), while in obese subjects, submucosal eosinophils were increased and correlated with blood eosinophils (r = 0.515) but not with sputum eosinophils (r = 0.276) [49].

In a study of bariatric surgery patients with and without asthma (N = 27 and 39, respectively), there were no changes 12 months post-operatively in submucosal eosinophils, neutrophils, CD20+ B cells, macrophages, CD4+ T cells, or CD8+ T cells, though the number of mast cells was found to be decreased in asthmatics [31]. In another study of 22 obese, severe asthmatics undergoing weight loss surgery, there were also no changes in sputum cell analysis (percentage of eosinophils, neutrophils, lymphocytes, macrophages, and epithelial cells), or serum IgE level at 6 months post-operatively [34]. In a cohort of obese women with and without asthma (N = 11 and 15, respectively), followed for 12 months after bariatric surgery, although BAL cytokines were overall similar in asthmatics and controls, BAL IL-8 and MCP-1 levels were higher in asthmatics at 12 months compared to their baseline [32]. In contrast, serum IL-6 and IL-8 were significantly lower in asthmatics at 12 months compared to baseline, while airway macrophages spontaneously produced more TNF-α, IL-6, IL-1β, and IL-10 at 12 months in the asthma group compared to baseline and controls. It is interesting that while weight loss studies have not demonstrated changes in sputum or tissue eosinophils, as discussed earlier, they have shown reduced airway hyperresponsiveness. These findings underscore the complex association between body weight and asthma, whose pathophysiology remains poorly understood. Furthermore, additional work suggests that obesity may reduce the sensitivity of biomarkers of type 2 inflammation, and that lower thresholds may be required in this patient population [50].

In the future, further study of approved anti-IL-5 biologic therapies may help clarify eosinophils’ role in obesity. Though data is limited at present, a study of 58 adult-onset asthma subjects prescribed anti-IL-5 therapy (N = 29 mepolizumab, N= 16 reslizumab, and N = 6 benralizumab) demonstrated a mean reduction in BMI by 1.0 ± 3.4 in the overall cohort and by 1.7 ± 3.8 in the obese group [51]. Given other modifiers of this association, such as reduced corticosteroid use or increased exercise capacity on therapy, more inquiry is required to determine whether type 2 inflammation directly contributes to obesity. 

### 4.3. Type 2 Innate Lymphoid Cells and Alarmins

Increasing evidence suggests that ILC2, which respond to epithelium-derived cytokines without the use of antigen-specific receptors, play an important role in allergic airway inflammation as signal amplifiers [52,53,54,55]. Prior to recuitment of the adaptive immune response manifested in T cells, local ILC2 are activated by “alarmins” secreted by damaged epithelial cells, such as IL-25, IL-33, and thymic stromal lymphopoietin (TSLP) [52]. ILC2 subsequently produce their own type 2 cytokines to influence and bolster incipient T cell immunity and to promote a predominant type 2 response that plays a key role in allergic asthma [56]. 

Multiple human studies have shown that ILC2 could be a superior surrogate marker for eosinophilic airway inflammation. In a cross-sectional study of 25 severe eosinophilic asthmatics, 19 mild atopic asthmatics, and five controls, a greater number of ILC2 were detected in both blood and sputum samples from severe asthmatics compared to mild asthmatics [57]. Individuals with uncontrolled airway eosinophilia (>3% sputum eosinophils) had higher numbers of IL-5 and IL-13 producing ILC2 compared to those with well-controlled airway eosinphilia (<3%) [57]. Another cross-sectional study of eosinophilic and non-eosinophilic asthmatics (n = 126) showed that the percent ILC2 in peripheral blood provided the strongest correlation with sputum eosinphil counts—better than IgE, blood eosinophils, or FeNO [58]. In BAL samples, asthmatics had higher levels of IL-33, and levels correlated with severity of asthma as judged by airway flow and asthma control test survey scores [59]. Additionally, the presence of asthma and level of IL-33 were associated with increased frequency of ILC2 cells in BAL [59]. 

Though limited, there are data relating ILC2 to obesity. In one study, the prevalence of ILC2 in subcutanous, white adiose tissue was decreased in obese samples versus non-obese samples [60]. In a cross-sectional study examing 87 subjects grouped into overweight/obese asthma (n = 20), normal weight asthma (n = 21), overweight/obese non-asthma (n = 25), and normal weight non-asthma (n = 21), the hauthors found reduced CD45RA+ ILC2, but increased CD45RO+ “inflammatory” ILC2 in obese asthmatics compared with normal weight asthmatics [61]. Notably, CD45RO+ ILC2 are known to be steroid resistant. Overall, however, human studies remain insufficient to establish a diffential role for ILC2 in obese asthmatics at this time [62].

Even though biologics, such as mepolizumab (anti-IL-5) and omalizumab (anti-IgE), are designed to block the type 2 pathyway [63], clinical data have shown that they are not effective in about half of allergic asthmatics [64,65]. However, alarmins, including IL-25, IL-33, and TSLP, derived from airway epithelial cells, have received increasing focus as upstream targets to block initial type 2 reactions and improve asthma control [64,65,66]. Since alarmins trigger both innate and adaptive immune responses, alarmin mitigation in allergic asthma represents a promising therapeutic prospect [66,67]. In fact, randomized, placebo-controlled trials have already shown that targeting alarmins is effective in controlling asthma. Most prominently, a phase 3 clinical trial testing tezepelumab, a human monoclonal blocking TSLP, reported improved frequency of asthma exacerbations, lung function, asthma control, and quality of life [68]. Additionally, a phase 2 trial of itepekimab, a monoclonal against IL-33, reported lower frequency of asthma exacerbation [69]. Astegolimab, a human monoclonal targeting IL-33 receptor, also showed reduced frequency of asthma exacerbation, an effect independent of blood eosinophil count [70]. Although more investigation is required, it would be particularly advantageous to develop asthma therapeutics that are effective independent of blood eosinophil counts [71]. As above, type 2 pathology, including eosinophilia, often does not predominate in obese asthmatics. In addition to providing therapeutic benefit, use of these drugs in patients with and without eosinophilia may help to expand our understanding of the unique pathophysiology in obese asthmatics. 

There are also some reports regarding an association between obesity and alarmins. A cross-sectional analysis of 127 adults showed that serum IL-33 is increased in obese adults [72]. In a 6-month observational study of 30 obese patients who underwent bariatric surgery and 15 non-obese, age-matched controls, the TSLP level was lower in obese subjects compared to the control group before and after the surgery, while no difference was observed in the other serum biomarkers tested—IL-25, IL-33, and IL-33 receptor—and there was no significant change detected after surgery [73]. Additionally, baseline TSLP was positively correlated with HbA1c, a biomarker for glucose metabolism, while accounting for age, gender, BMI, serum glucose, and lipid levels [73]. Lastly, in a cohort study of type 2 diabetes and control subjects, gene expression of IL-33 in subcutaneous tissue was higher in type 2 diabetes than controls and was associated with insulin resistance [74]. Although alarmins are known to be associated with obesity, to our knowledge, there are unfortunately no human studies specifically exploring the role of alarmins in obese asthmatics.

### 4.4. FeNO

FeNO is clinically used to assess allergic airway inflammation in asthma [75] and has also been used to diagnose asthma and predict steroid responsiveness [76]. A study of children aged 6 to 17 years (52 obese asthmatics, 49 normal-weight asthmatics, 51 obese non-asthmatics, and 42 normal-weight non-asthmatics) reported a positive association between weight and FeNO only in the non-asthmatic group [77], and, in another study of 447 children, BMI was not associated with FeNO levels [78]. In a subgroup of the NHANES survey (N = 789), those who were obese and had a high waist circumference tended to have lower FeNO levels, but the associations were statistically insignificant [41]. In contrast, a study of non-asthmatic, non-smoking students (mean age 20 years) found FeNO was positively associated with BMI, waist and hip circumferences, waist-hip ratio, and visceral fat percentage [79]. Building upon these conflicting results, several studies suggested more complex relationships. A cross-sectional study of 929 adolescents measured lower FeNO in both the underweight and overweight compared to the normal weight group, accounting for asthma status, providing an inverted U-shaped curve relating BMI and FeNO [80]. A 12-month cohort of well-controlled asthmatic adults found that the high visceral fat group had higher sputum IL-6 and IL-8 levels, but lower FeNO and AEC compared to the low visceral fat group [81]. An analysis of 1607 middle-aged adults found that allergic asthma could be positively correlated with FeNO and AEC, but only when those asthmatic subjects were currently non-smokers [82]. A behavioral intervention comparing weight loss programs with and without exercise in obese asthmatic adults (mean total body weight change −6.8% and −3.1%, respectively) reported that the exercise group showed lower FeNO, IL-4, CCL2, TNF-α, and leptin levels, while increasing vitamin D, IL-10, and adiponectin levels compared to the weight loss without exercise group [83]. While studies targeting general populations have shown positive associations between FeNO and adiposity, the clinical utility of FeNO measurement in non-asthmatics has not been established, and even in asthmatics, the association is controversial. Modifiers of FeNO levels, such as asthma severity, degree of control, and inhaled exposures, may confound the associations between body weight and FeNO level. 

## 5. Is Obesity Associated with Dysfunctions of Airway Epithelial Cells?

Airway epithelial cells protect the human body from inhaled environmental exposures [84], and damage to epithelial cells renders the airway susceptible to allergen-induced inflammation, predisposing to asthma development and progression [3,85,86]. Conversely, asthmatic inflammation can destroy epithelial tight junctions and cilia, while expanding smooth muscle and goblet cells, thickening the basal membrane, inducing subepithelial fibrosis, and depositing extracellular debris [29,87,88,89,90,91]. Studies have also shown that obesity is independently associated with damage to airway epithelial cells [92], with the proposed mechanism to involve metabolic oxidative stress, triggering persistent immune cell recruitment and inflammation. This phenomenon is most prominent in patients with both asthma and obesity, where a positive feedback loop is established between airway epithelial injury and inflammation [93]. 

Regarding a specific metabolic pathway, paraoxonase 2 (PON2) has been considered, given its role in mitigating mitochondrial-derived oxidative stress. One study compared PON2 levels in human bronchial epithelial cells obtained via bronchoscopy from lean controls (N = 10), lean asthmatics (N = 8), obese controls (N = 11), and obese asthmatics (N = 8), finding that cells derived from obese asthmatics exhibited lower PON2 levels than those from lean asthmatics and lean controls [94]. Perhaps hinting at another mechanism, adipose tissue-derived adiponectin was found to be negatively associated with the chemokines CCL2 and CXCL1 but positively associated with CCL20, CXCL8, and IL-6 in a dose-dependent manner in human bronchial epithelial cells [95]. Adiponectin was also shown to inhibit TNF-α-induced production of CXCL1 and CCL2; however, adipose-derived leptin, chemerin, and visfatin did not alter cytokine production. In another study, airway epithelial cells produced more CXCL1, CXCL2, and IL-8 when exposed to adipose tissue-conditioned media derived from allergic obese asthmatics, as compared with obese non-asthmatics or non-allergic asthma groups [96]. The authors additionally collected serial plasma samples from obese asthmatics (both type 2-high and -low) and obese non-asthmatics who underwent bariatric surgery. When airway epithelial cells were exposed to plasma from the obese non-asthmatic group, IL-8, CCL20, G-CSF, and IL-6 levels decreased, with a similar trend in the obese type 2-low asthma group. However, the obese type 2-high asthma group demonstrated a decrease in IL-8 at 6 months that returned to baseline at 12 months, with increased CCL20 at 12 months, and G-CSF decreased at 3 and 6 months but increased at 12 months [96]. These findings suggest that adipokines may alter airway epithelial function and that weight loss may not fully resolve inflammation in patients with obesity and asthma. 

It may be worth mentioning that studies have also examined nasal epithelium, though bronchial epithelium is arguably more relevant to asthma. In a combined analysis of two cohort studies conducting RNA sequencing on nasal epithelium from asthmatics aged 9–20 years (N = 235) and 6–16 years (N = 66), respectively, it was found that pathways for IFN signaling, and innate and adaptive immune responses were down-regulated in overweight/obese subjects, while pathways related to ciliary structure or function were up-regulated [97]. 

## 6. Discussion

We reviewed the extant scientific literature regarding human studies investigating the association between excess body weight and asthma, where obesity is an established risk factor for developing asthma and asthma exacerbation [98]. Some of the strongest data involve airway hyperreactivity, where observational and interventional studies support the theory that obesity is associated with an increased risk of airway hyperreactivity, though the underlying pathophysiology remains unclear. In general, when parameters of allergic airway inflammation are used to evaluate the relationship between obesity and asthma, studies often conflict in their findings, likely influenced by confounding variables such as socioeconomics, hygiene, and exercise. Toward this notion, in vitro and in vivo models focusing on a single or few pathologic pathways have successfully shown significance of their pathologic pathways; however, these findings are often not supported or confirmed in human studies. Several limitations are variabilities in human characteristics, presence of modifiers, complex biological pathways limiting our ability to fully disentangle the role of a specific pathway, and inability to control potential confounders and variabilities in humans.

Longitudinal studies examining people with severe asthma have shown that type 2-high and type 2-low phenotypes can transition over time, and that most severe asthma patients exhibit heightened eosinophilic inflammation and other type 2 biomarkers over a roughly 10-year period; however, this signal may weaken even as their clinical asthma persists [12,13,14]. This suggests that both pathways likely coexist and simultaneously contribute to asthma pathology. Since asthma initially develops after sensitization to aeroallergens and recurrent exposure-induced hyperreactivity, the type 2 pathway may be actively involved, even in the pathophysiology type 2-low asthmatics. In the future, expanding the indication for type 2-targeted therapies to obese asthmatics with type 2-low phenotypes may help to reveal the contribution of the type 2 pathway in these patients. Targeted therapy for weight loss, such as glucagon-like peptide-1 receptor (GLP1R) agonists, may also help us to understand the role of obesity in asthma. GLP1R agonists are increasingly used to treat obesity and have been proposed for treating asthma [99,100]. Data collected from the initial pharmacological trials of these medications could help further clarify the role of body weight in asthma.

A more sophisticated methodology will be required to assess the type 2 pathway and asthma immunology generally. As discussed above, the correlation between eosinophil counts in blood, sputum, BAL, and bronchial tissue is relatively weak, and no consensus exists on which eosinophil marker is the strongest predictor of asthma severity. Furthermore, these quantitative measures do not reflect the functionality of eosinophils or their influence on clinical disease. Studies measuring cytokines and cell counts from various anatomical approaches have reported conflicting findings that resist the development of a comprehensive theory to model asthma pathology. It is possible that the current scope of the current study has been too narrow and that we have failed to consider additional important variables. If so, increased pursuit of broad-based approaches in transcriptomics, proteomics, and metabolomics may be required to fill in the gaps. 

Lastly, a lack of longitudinal cohorts presents a significant limitation in our understanding of asthma, given its chronicity and heterogeneity. Evidence suggests that asthma pathophysiology and phenotypes often shift within the lifetime of patients, and additional longitudinal cohort studies would aid us in explaining how and why asthma evolves over time.

## 7. Conclusions

Existing evidence suggests that asthma and obesity are associated with each other, and that immunological pathways involving both type 2-high and type 2-low phenotypes are involved in that interaction. Despite these findings, studies have yet to elucidate the underlying pathophysiological processes that explain the association. Progress has been difficult, as both asthma and obesity are complex chronic diseases marked by significant heterogeneity. However, future data from large-scale population-based longitudinal studies with comprehensive immunologic readouts may guide our understanding of the biological pathways that connect asthma and obesity. 

## Data Availability

No new data were created or analyzed in this study.
